# Memantine increases NMDA receptor level in the prefrontal cortex but fails to reverse apomorphine-induced conditioned place preference in rats

**DOI:** 10.3934/Neuroscience.2018.4.211

**Published:** 2018-09-21

**Authors:** Ziphozethu Ndlazi, Oualid Abboussi, Musa Mabandla, Willie Daniels

**Affiliations:** 1 Department of Physiology, School of Laboratory of Medicine and Medical Sciences, University of KwaZulu-Natal, Durban, 4001, South Africa; 2 Institute of Academic Anaesthesia, Division of Neuroscience, Ninewells Hospital and Medical School, University of Dundee, Dundee, UK; 3 School of Physiology, Faculty of Health Sciences, University of the Witwatersrand, Johannesburg, South Africa

**Keywords:** apomorphine, memantine, conditioned place preference, NMDA receptors

## Abstract

Studies have shown that inflammation and neurodegeneration may accompany the development of addiction to apomorphine and that the glutamate NMDA receptor antagonist, memantine, may be neuroprotective. The similarity between apomorphine and dopamine with regard to their chemical, pharmacological and toxicological properties provided a basis for investigating the mechanism of action of the former agent. In this study, we investigated whether memantine would suppress apomorphine-seeking behavior in rats subjected to apomorphine-induced place preference conditioning, through modulation of NMDA receptors in the prefrontal cortex. Repeated apomorphine (1 mg/kg) treatment induced conditioned place preference (CPP) and had no significant effect on NMDA receptor levels in the prefrontal cortex. Prior treatment with memantine (5 mg/kg or 10 mg/kg) increased the levels of NMDA receptors in the prefrontal cortex but did not suppress CPP induced by apomorphine. These data give further support to the addictive effect of apomorphine and demonstrate that blockade of NMDA receptors by memantine is unable to suppress apomorphine-seeking behavior.

## 1. Introduction

The risk of development of addictive behavior as a side effect of many pharmacological therapies for psychiatric disorders, has increased in the last two decades and represents a serious problem in our societies today [1]. Among these drugs is apomorphine, a non-narcotic derivate of morphine, which acts as a dopamine receptor agonist to produce psychostimulant-like effects [2]. Apomorphine is prescribed as a treatment for alcohol and narcotic abuse, it is advocated as an antiparkinsonian agent [3] and has recently been proposed as a potential treatment for Alzheimer's disease [3],[4]. However, accumulated clinical evidence indicates that chronic treatment with apomorphine leads to addiction [5]. Indeed, a series of animal studies have supported a rewarding effect of apomorphine when tested in a conditioned place preference (CPP) paradigm [2],[6],[7]. These observations are not surprising as apomorphine and dopamine share structural and functional similarities. Apomorphine is in many respects considered a lipophilic dopamine with a catechol moiety [8]. Similar to dopamine, apomorphine can undergo rapid auto-oxidation to quinones [9], generating reactive chemical species that may be toxic to neurons [10].

Thus, the development of pharmacological agents that may suppress the rewarding effects of apomorphine, is important for its continuing therapeutic use. In this regard, one potential target system is the glutamatergic system. Glutamate is a neurotransmitter central to learning and neuroplasticity, phenomena that have been positively linked to addiction processes within the limbic-cortico-striato-cortical circuit [11],[12]. Morphological studies support the existence of axon-axon synapses of glutamate and dopamine neurons in the medial PFC, ventral striatum and NAcc providing evidence at the synaptic level for interactions between these two neurotransmitters [13]. Collectively these findings provide both an anatomical and functional basis for a role for glutamate in addiction and therefore support initiatives that investigate its contribution to the development of substance use disorders.

Memantine, a non-competitive glutamate NMDA receptor antagonist has been shown to inhibit the expression of CPP induced in animals with several drugs that are abused by humans such as cocaine and morphine at doses of 10 mg/kg, i.p. [14]. It has also been demonstrated that memantine at 5 mg/kg, i.p. protects inflammation-related degeneration of dopamine neurons [14],[15]. These beneficial effects of memantine might be related to the fact that dopamine release in appetitive behaviors and drug abuse, requires glutamatergic input, activation of NMDA receptors, an opening of high-threshold calcium currents, and finally activation of calcium-activated potassium currents to terminate the burst of neuronal firing [16].

The aim of the present study was therefore to investigate the potential beneficial effect of memantine in apomorphine-induced reward-related behavior. We analyzed the rewarding effects of apomorphine using a rat place preference conditioning paradigm. We also assessed the impact of apomorphine and memantine on the glutamatergic system by determining its effects on the level of NMDA receptors in the prefrontal cortex. We focussed on this brain region as it appears to be central to the effects of both dopamine and glutamate in their regulation of emotional responses, cognitive control, and executive functions [17].

## 2. Materials and methods

### 2.1. Animals

A total of 40 adult male Sprague-Dawley (SD) rats weighing between 180–250 g were used in this study. The animals were housed in groups of four per cage, with free access to food and water. The animals were kept at a constant temperature (21–23 °C) under a 12/12 h light-dark cycle (lights on at 06:00). Animals were tested during the light phase. Before starting the experiment, rats were accustomed to various handling procedures in order to nullify the psychological effect of environment.

### 2.2. Treatment procedure

**Figure 1.**
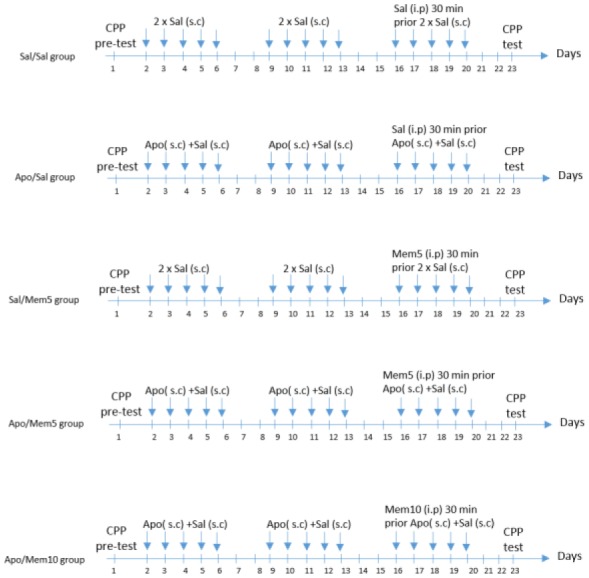
Timeline of the 3 phases of the conditioned place preference protocol (Apo: apomorphine (1 mg/kg); Sal: saline (Nacl0.9%); Mem5: Memantine (5 mg/kg); Mem10: Memantine (10 mg/kg)).

The rats were divided into 5 groups (n = 8 each) and received the following treatments: Saline group (Sal/Sal) was given a daily subcutaneous injection of saline, twice a day, for 5 days followed by 2 days of withdrawal for 3 successive weeks. During the third week, the subcutaneous injections were preceded (30 min) by an i.p. injection of saline. The apomorphine group (Apo/Sal) was given a daily subcutaneous injection of 1 mg/kg of apomorphine and saline for 5 days followed by 2 days of withdrawal for 3 successive weeks. During the last week saline was injected i.p. 30 min prior to apomorphine administration. The memantine (Mem5/Sal) group was given a daily subcutaneous injection of saline for 5 days followed by 2 days of withdrawal for 3 successive weeks. During the last week, memantine 5 mg/kg was injected i.p. 30 min prior to saline. The two apomorphine-memantine (Apo/Mem5 and Apo/Mem10) groups were given a daily subcutaneous injection of 1 mg/kg of apomorphine for 5 days followed by 2 days of withdrawal for 3 successive weeks. During the third week, memantine 5 mg/kg or 10 mg/kg was given i.p. 30 min prior to apomorphine. The protocol for the administration of the various drugs is depicted in Figure 1.

All experimental procedures were approved by the Animal Ethics Research Committee of the University of KwaZulu-Natal (Ethics approval number 075/11; 081/12) and were in accordance with the National Institutes of Health Guide for the Care and Use of Laboratory Animals (NIH Publications No. 85-23, revised 1985).

### 2.3. Drugs

Apomorphine and memantine hydrochloride (Sigma-Aldrich, Missouri, USA) were dissolved in saline and injected subcutaneously and intraperitoneally respectively. Drug solutions were freshly prepared before each experiment. Control animals were injected with sterile saline (0.9% NaCl).

### 2.4. Conditioned place preference paradigm

Place conditioning was conducted in a two-compartment apparatus (23 × 23 × 23 cm^3^ each compartment) divided by a sliding partition. The compartments provided distinct contexts, with one compartment having horizontal and vertical white striped walls with a checked white pattern and net-like white mesh floor (A). The other compartment had black plain walls and a black plain smooth floor. The testing paradigm involved three phases [18]:

(i) A pre-test phase: This phase took place on day one of the experiments, where each rat was placed individually into the apparatus for 15 min and was allowed free access to both compartments. The time spent in each compartment was recorded to determine the naturally preferred chamber for the animal.
(ii) Conditioning phase: During five consecutive days, twice per day, animals were treated with apomorphine 1 mg/kg or saline 0.9% and subsequently paired with either the white or black compartment, where they were allowed to roam for 30 min [19],[20]. The control group was paired with saline in both compartments while apomorphine-treated animals received saline in the preferred compartment and apomorphine in the less preferred compartment. This five-day schedule was repeated over a three-week period to induce place preference for apomorphine. During the third week, for five consecutive days, apomorphine-treated animals received saline 0.9% or memantine at 5 or 10 mg/kg [21], 30 min prior to apomorphine injections in order to block the expression of CPP induced by apomorphine (Figure 1).
(iii) Postconditioning: This phase was carried out on day 24, in a drug-free state. The animal was once again placed in the place preference apparatus and allowed free access to both compartments for 15 min.

Conditioning scores were calculated as the time spent in the initially least preferred compartment.

### 2.5. Tissue collection

On day 24, animals were decapitated and their prefrontal cortex tissue was harvested, snap frozen in liquid nitrogen and stored in a bio-freezer at −75 °C. The tissue was later used for biochemical analysis.

### 2.6. Determination of glutamate receptor density in the frontal cortex using an enzyme-linked immunosorbent assay (ELISA)

A commercially available rat glutamate receptor subunit zeta 1 (GRINL1A) ELISA kit (CUSABIO^®^) was used for the quantitative determination of glutamate receptor levels in frontal cortex of drug-treated and control animals. Twenty-five mg of rat prefrontal cortex tissue was rinsed, sonicated in 1 ml of phosphate-buffered saline, and stored overnight at −20 °C. After two freeze-thaw cycles were performed to rupture the cell membranes, the homogenates were centrifuged for 5 min at 2000 x g, at 4 °C. Supernatants were removed and assayed immediately following the manufacture's protocol. Basically, the protocol entailed preparation of the reagents, samples and standards as instructed in the protocol, addition of 100 µl standard or sample into each well (assay plate, 12 × 8 coated microwells) to be incubated at 37 °C for 2 hrs. Liquid was then removed from each well and 100 µl Biotin-antibody (1x) was added to the wells and incubated for an hour at 37 °C, this liquid was then removed through aspiration and the plate was washed three times with the wash buffer (200 µl) using a multi-channel pipette and was left to stand for 2 min followed by complete removal of the liquid by aspiration and inversion of the plate, blotting it on paper towel. One hundred µl of HRP-avidin was added to the wells and the plate was covered with an adhesive strip and again was incubated for an hour at 37 °C followed by the aspiration/wash process for five times. Ninety µl TMB Substrate was added to each well and the plate was covered with foil to protect it from light and incubated for 20 min at 37 °C. After this step was completed a Stop Solution was introduced to each well and the plate was gently tapped to ensure thorough mixing. The optical density of each well was subsequently determined within 5 min using a microplate reader that was set to 450 nm.

### 2.7. Statistical analysis

All results are reported as the mean ± SEM (standard error of mean). Data were analyzed using GraphPad Prism (version 5, San Diego, California, USA). The Shapiro-Wilk test revealed that the CPP data were normally distributed. A repeated measures ANOVA was used to examine memantine effects on apomorphine-induced CPP (pre- vs post-treatment), followed by the Bonferroni multiple comparison posthoc test. The NMDA receptor data were not normally distributed and as a result group differences in NMDA receptor levels were expressed as medians and interquartile range and analyzed by the Kruskal-Wallis test (non-parametric test) followed by Dunn's multiple comparison test. The levels of statistical significance were set at *p* < 0.05.

## 3. Results

### 3.1. Effect of memantine on apomorphine-induced place preference

**Figure 2.**
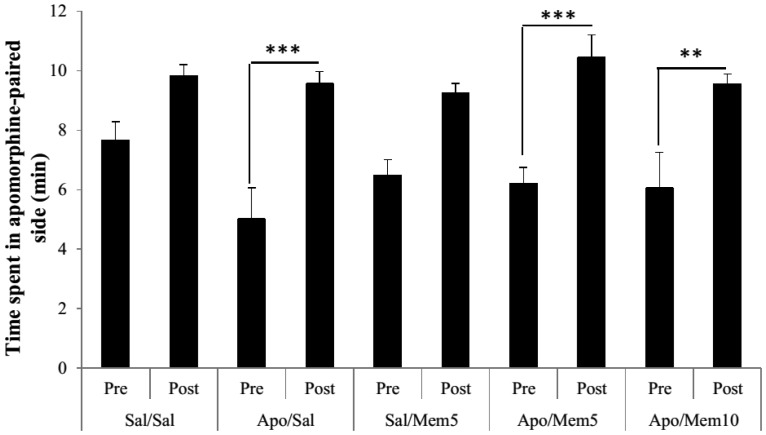
Effect of memantine on apomorphine-induced place-preference in rats.Data are means ± S.E.M. **p < 0.01, ***p < 0.001, pre-conditioning vs post-conditioning (Bonferroni post hoc test) (n = 8 per group).Apo: apomorphine (1 mg/kg); Sal: saline (Nacl0.9%); Mem5: Memantine (5 mg/kg); Mem10: Memantine (10 mg/kg).

As depicted in Figure 2, repeated measures ANOVA with time as repeated measure and treatment as factor revealed a significant treatment effect (F(9,80) = 10.55, *p* < 0.001). The Bonferroni test for differences between time spent in the apomorphine-associated compartment during the pre-treatment and post-treatment sessions showed no significant differences in saline (Sal) and memantine 5 mg (Mem5) treated groups (*p* > 0.05). However, a highly significant difference between time spent in the apomorphine-associated compartment during the pre- and post-treatment sessions was revealed in animals undergoing chronic apomorphine treatment (*p* < 0.001) which suggests successful conditioning with apomorphine. The treatment of apomorphine-conditioned animals with memantine at 5 or 10 mg/kg did not change the time spent in the apomorphine-paired compartment (pre- vs post-treatment *p* = 0.0103 and *p* = 0.0332 respectively) suggesting that both 5 and 10 mg/kg doses of memantine did not block the rewarding effects of apomorphine.

### 3.2. Effect of memantine on prefrontal cortex NMDA receptor levels in animals undergoing chronic apomorphine treatment

**Figure 3.**
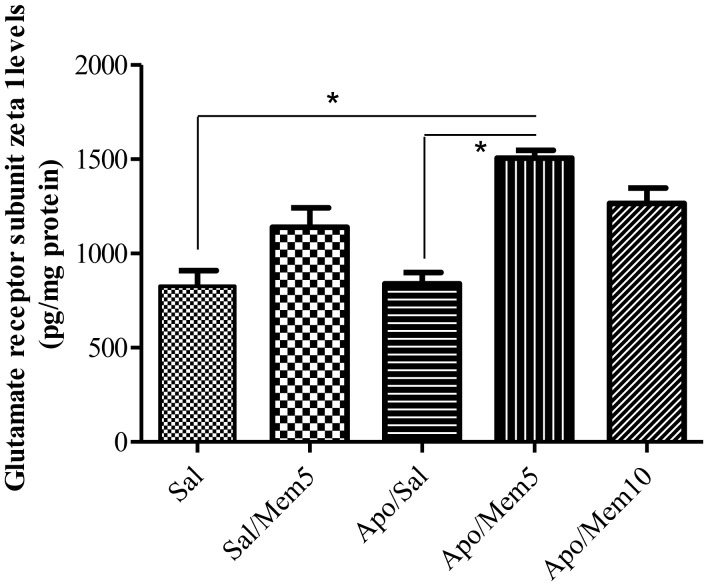
Effect of memantine (Mem) on NMDA receptor levels in the prefrontal cortex of animals subjected to chronic apomorphine (Apo) treatment. The median was 832.5 (interquartile range: 660.9–980.1) for Sal/Sal, 1136 (894–1390) for Sal/Mem5, 791.2 (754.3–1019) for Apo/Sal, 1479 (1436–1629) for Apo/Mem5 and 1246 (1092–1478) Apo/Mem10. Data are presented as mean ± SEM. *p < 0.05 compared with control group (Sal/Sal) (Dunn's Multiple comparison test) (n = 4 per group).

We further investigated glutamate NMDA receptor levels in the prefrontal cortex of rats treated with memantine and subjected to apomorphine-induced CPP (Figure 3). The non-parametric Kruskal-Wallis test revealed a main effect of treatment on NMDA receptor levels (*p* = 0.0045, n = 4 rats/group), Dunn's multiple comparison test showed that chronic apomorphine treatment induced no significant effect compared to control group (*p* > 0.05), however memantine at 5 mg/kg induced a significant increase in NMDA receptor density (*p* < 0.05).

## 4. Discussion

This study assessed the potential anti-addictive effect of memantine in male rats undergoing chronic exposure to apomorphine, as well as the expression of NMDA receptors in the prefrontal cortex. Importantly, chronic exposure to apomorphine-induced CPP as expected but did not alter the expression of NMDA receptors. On other hands, memantine administered prior to apomorphine for 5 days prior to testing, failed to block apomorphine-induced CPP. Memantine did, however, upregulate NMDA receptors in apomorphine-treated rats.

Our results are consistent with previous behavioral studies indicating that apomorphine has reinforcing properties when administrated chronically at comparable doses (0.5, 1, 2.5 mg/kg) [22]–[24]. These properties are associated with direct stimulation of dopaminergic receptors D1 and D2 [5],[23]. The effect of apomorphine, as well as all drugs of abuse, on the dopaminergic system, is usually paralleled by adaptive changes in the glutamatergic system [25],[26]. These changes include increased responsiveness and expression of glutamatergic receptors, in particular, NMDA receptors expressed on dopaminergic neuron terminals in the prefrontal cortex [27]. Thus several studies suggested NMDA receptor antagonists as a potential treatment for the abuse of substances such as amphetamine, cocaine, and alcohol [15],[28],[29]. Indeed, NMDA receptor blockade has been shown to interfere with the development, maintenance and the expression of addiction-like behavior in animal models of morphine and alcohol addiction [30], However, under our experimental conditions, chronic exposure to apomorphine did not affect NMDA receptor levels in the prefrontal cortex which suggests that altered expression of NMDA receptors in the prefrontal cortex may not be a prominent feature of the conditioned response to apomorphine. Furthermore, it might be argued that the discrepancies between our study and previous findings could have been due to the duration of the treatment, or the duration of abstinence between the end of chronic treatment and behavioral testing. Thus further studies on the effects of long-term apomorphine exposure in the corticostriatal pathways are needed to understand how changes in dopaminergic transmission may affect glutamatergic transmission to induce drug-seeking behavior. On the other hand, in accordance with the well-established effect of NMDA receptor antagonists in inducing NMDA receptor upregulation in animal models of drug addiction and in cultured cortical neurons [31], treatment with memantine increased NMDA receptors in the prefrontal cortex of apomorphine-conditioned rats but not in control rats. This may suggest that apomorphine treatment may have altered the glutamatergic system to make it more susceptible to the effects of memantine. Interestingly, this effect of memantine on NMDA receptor expression had no influence on apomorphine-induced CPP. The lack of effect of apomorphine on NMDA receptor levels in the prefrontal cortex is supported by the inability of memantine-induced up-regulation of NMDA receptors to alter apomorphine-seeking behavior. However, since memantine was absent during the first 2 weeks of conditioning as well as during memory recall in the post-treatment test of place preference, it may be possible that once CPP is established by apomorphine, manipulation of NMDA receptors by the administration of memantine has little consequence on drug-seeking behavior. It is noteworthy that the memantine-mediated increase in NMDA receptor levels also did not worsen drug-seeking behavior in animals that were treated with apomorphine. Thus, in contrast to the proposed therapeutic effect of NMDA receptor blockade by memantine [32]–[34], our study showed that up-regulation of the NMDA receptors by memantine after the establishment of CPP by apomorphine had minimal impact on the reversal of CPP. The fact that memantine did increase NMDA receptors in the prefrontal cortex indicates that its beneficial effects may be limited to certain specific circumstances only, such as the expression and maintenance of morphine, alcohol, and cocaine dependencies [35]–[37]. Our results, therefore, caution against the widespread use of memantine as a pharmacological intervention, especially in view of its ability to increase the number of NMDA receptors. Elevations in NMDA receptors could cause neural cells to be more vulnerable to glutamate excitotoxicity, since NMDA receptor up-regulation may result in an excessive entry of calcium, triggering a series of cytoplasmic and nuclear processes such as loss of mitochondrial membrane potential, which ultimately can lead to neuronal cell death [38].

Taken together, our data suggest that maintenance of apomorphine-induced CPP does not require upregulation of NMDA receptor expression in the prefrontal cortex, but may depend on indirect or compensatory mechanisms implicated in apomorphine-induced drug seeking.

In conclusion, these findings provide further support for the drug-seeking-like effect of apomorphine and suggest that the prescription of memantine as a possible treatment for addiction must be done with caution. However, further studies are necessary to explore the functional consequences of memantine-induced increases in NMDA receptor expression.
